# 2-[2-(Methyl­sulfon­yl)eth­yl]isoindoline-1,3-dione

**DOI:** 10.1107/S160053680902580X

**Published:** 2009-07-18

**Authors:** Qiong Tang, Qi Feng, Jian Xu, Cheng Yao

**Affiliations:** a Department of Applied Chemistry, College of Science, Nanjing University of Technology, Xinmofan Road No. 5 Nanjing, Nanjing 210009, People’s Republic of China

## Abstract

In the mol­ecule of the title compound, C_11_H_11_NO_4_S, the isoindoline ring system is almost planar with a maximum deviation of 0.008 (3)Å. In the crystal structure, inter­molecular C—H⋯O inter­actions link the mol­ecules into a three-dimensional network. π–π contacts between the isoindoline rings [centroid–centroid distances = 3.592 (1) and 3.727 (1) Å] may further stabilize the structure.

## Related literature

For a related structure, see: Kilburn *et al.* (2007 ▶). For bond-length data, see: Allen *et al.* (1987 ▶).
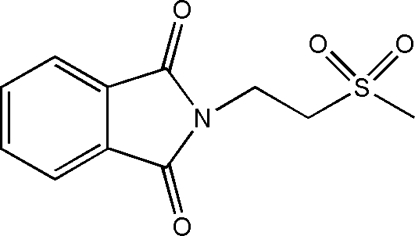


## Experimental

### 

#### Crystal data

C_11_H_11_NO_4_S
*M* *_r_* = 253.27
Monoclinic, 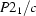

*a* = 7.6030 (15) Å
*b* = 17.766 (4) Å
*c* = 8.9940 (18) Å
β = 112.31 (3)°
*V* = 1123.9 (5) Å^3^
*Z* = 4
Mo *K*α radiation
μ = 0.29 mm^−1^
*T* = 294 K
0.20 × 0.10 × 0.10 mm

#### Data collection

Enraf–Nonius CAD-4 diffractometer
Absorption correction: ψ scan (North *et al.*, 1968 ▶) *T*_min_ = 0.944, *T*_max_ = 0.972
2182 measured reflections
2027 independent reflections
1567 reflections with *I* > 2σ(*I*)
*R*_int_ = 0.030
3 standard reflections frequency: 120 min intensity decay: 1%

#### Refinement

*R*[*F* ^2^ > 2σ(*F* ^2^)] = 0.048
*wR*(*F* ^2^) = 0.144
*S* = 1.01
2027 reflections
154 parameters
H-atom parameters constrained
Δρ_max_ = 0.23 e Å^−3^
Δρ_min_ = −0.35 e Å^−3^

### 

Data collection: *CAD-4 Software* (Enraf–Nonius, 1989 ▶); cell refinement: *CAD-4 Software*; data reduction: *XCAD4* (Harms & Wocadlo, 1995 ▶); program(s) used to solve structure: *SHELXS97* (Sheldrick, 2008 ▶); program(s) used to refine structure: *SHELXL97* (Sheldrick, 2008 ▶); molecular graphics: *ORTEP-3 for Windows* (Farrugia, 1997 ▶); software used to prepare material for publication: *SHELXL97* and *PLATON* (Spek, 2009 ▶).

## Supplementary Material

Crystal structure: contains datablocks I, global. DOI: 10.1107/S160053680902580X/hk2730sup1.cif

Structure factors: contains datablocks I. DOI: 10.1107/S160053680902580X/hk2730Isup2.hkl

Additional supplementary materials:  crystallographic information; 3D view; checkCIF report

## Figures and Tables

**Table 1:** Hydrogen-bond geometry (Å, °)

*D*—H⋯*A*	*D*—H	H⋯*A*	*D*⋯*A*	*D*—H⋯*A*
C1—H1*A*⋯O3^i^	0.93	2.34	3.189 (5)	152
C11—H11*A*⋯O1^ii^	0.96	2.51	3.463 (4)	175

Symmetry codes: (i) 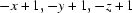; (ii) 
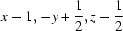
.

**Fig. 1.**
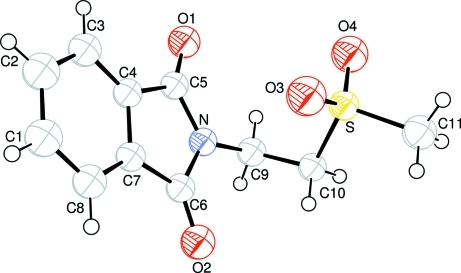
The molecular structure of the title molecule, with the atom-numbering scheme.

## supplementary crystallographic information

### Comment

The title compound is an important pharmaceutical intermediate, which is used in treatment of metabolic syndrome. As part of our studies in this area, we report herein the crystal structure of the title compound.

In the molecule of the title compound (Fig 1), the bond lengths (Allen *et al.*,  1987) and angles are within normal ranges. Rings A (N/C4-C7) and B (C1-C4/C7/C8) are, of course, planar and the dihedral angle between them is A/B = 0.61 (3)°. The isoindoline ring system is planar with a maximum deviation of -0.008 (3) Å for atom N.

In the crystal structure, intermolecular C-H···O interactions (Table 1) link the molecules into a three-dimensional network, in which they may be effective in the stabilization of the structure. The π–π contacts between the isoindoline rings, Cg1—Cg2^i^ and Cg2—Cg2^ii^ [symmetry codes: (i) 2 - x, -y, 2 - z, (ii) 1 - x, -y, 2 - z, where Cg1 and Cg2 are centroids of the rings A (N/C4-C7) and B (C1-C4/C7/C8), respectively] may further stabilize the structure, with centroid-centroid distances of 3.592 (1) and 3.727 (1) Å, respectively.

### Experimental

The title compound was prepared according to the literature method (Kilburn *et al.*,  2007). Crystals suitable for X-ray analysis were obtained by dissolving the title compound (0.1 g) in acetone (25 ml) and evaporating the solvent slowly at room temperature for about 7 d.

### Refinement

H atoms were positioned geometrically with C-H = 0.93, 0.97 and 0.96 Å for aromatic, methylene and methyl H atoms, respectively, and constrained to ride on their parent atoms, with U_iso_(H) = xU_eq_(C), where x = 1.5 for methyl H and x = 1.2 for all other H atoms.

### Crystal data

**Table:**

C_11_H_11_NO_4_S	*F*(000) = 528
*M**_r_* = 253.27	*D*_x_ = 1.497 Mg m^−^^3^
Monoclinic, *P*2_1_/*c*	Mo *K*α radiation, λ = 0.71073 Å
Hall symbol: -P 2ybc	Cell parameters from 25 reflections
*a* = 7.6030 (15) Å	θ = 9–13°
*b* = 17.766 (4) Å	µ = 0.29 mm^−^^1^
*c* = 8.9940 (18) Å	*T* = 294 K
β = 112.31 (3)°	Block, colorless
*V* = 1123.9 (5) Å^3^	0.20 × 0.10 × 0.10 mm
*Z* = 4	

### Data collection

**Table:**

Enraf–Nonius CAD-4 diffractometer	1567 reflections with *I* > 2σ(*I*)
Radiation source: fine-focus sealed tube	*R*_int_ = 0.030
graphite	θ_max_ = 25.3°, θ_min_ = 2.3°
ω/2θ scans	*h* = 0→9
Absorption correction: ψ scan (North *et al.*, 1968)	*k* = 0→21
*T*_min_ = 0.944, *T*_max_ = 0.972	*l* = −10→9
2182 measured reflections	3 standard reflections every 120 min
2027 independent reflections	intensity decay: 1%

### Refinement

**Table:**

Refinement on *F*^2^	Primary atom site location: structure-invariant direct methods
Least-squares matrix: full	Secondary atom site location: difference Fourier map
*R*[*F*^2^ > 2σ(*F*^2^)] = 0.048	Hydrogen site location: inferred from neighbouring sites
*wR*(*F*^2^) = 0.144	H-atom parameters constrained
*S* = 1.01	*w* = 1/[σ^2^(*F*_o_^2^) + (0.09*P*)^2^] where *P* = (*F*_o_^2^ + 2*F*_c_^2^)/3
2027 reflections	(Δ/σ)_max_ < 0.001
154 parameters	Δρ_max_ = 0.23 e Å^−^^3^
0 restraints	Δρ_min_ = −0.35 e Å^−^^3^

### Special details

**Table:**

Geometry. All e.s.d.'s (except the e.s.d. in the dihedral angle between two l.s. planes) are estimated using the full covariance matrix. The cell e.s.d.'s are taken into account individually in the estimation of e.s.d.'s in distances, angles and torsion angles; correlations between e.s.d.'s in cell parameters are only used when they are defined by crystal symmetry. An approximate (isotropic) treatment of cell e.s.d.'s is used for estimating e.s.d.'s involving l.s. planes.
Refinement. Refinement of *F*^2^ against ALL reflections. The weighted *R*-factor *wR* and goodness of fit *S* are based on *F*^2^, conventional *R*-factors *R* are based on *F*, with *F* set to zero for negative *F*^2^. The threshold expression of *F*^2^ > σ(*F*^2^) is used only for calculating *R*-factors(gt) *etc*. and is not relevant to the choice of reflections for refinement. *R*-factors based on *F*^2^ are statistically about twice as large as those based on *F*, and *R*- factors based on ALL data will be even larger.

### Fractional atomic coordinates and isotropic or equivalent isotropic displacement parameters (Å^2^)

**Table:**

	*x*	*y*	*z*	*U*_iso_*/*U*_eq_	
S	0.44021 (11)	0.29490 (4)	0.03299 (8)	0.0425 (3)	
O1	0.8805 (3)	0.29528 (10)	0.4407 (3)	0.0524 (6)	
O2	0.7221 (3)	0.50798 (11)	0.1325 (3)	0.0555 (6)	
O3	0.3992 (4)	0.33791 (14)	0.1503 (3)	0.0700 (7)	
O4	0.5068 (4)	0.21958 (12)	0.0776 (3)	0.0662 (7)	
N	0.8004 (3)	0.39124 (12)	0.2541 (3)	0.0394 (6)	
C1	0.7239 (5)	0.55296 (19)	0.6247 (4)	0.0582 (9)	
H1A	0.6981	0.5972	0.6679	0.070*	
C2	0.7703 (5)	0.4883 (2)	0.7187 (4)	0.0584 (9)	
H2A	0.7765	0.4901	0.8239	0.070*	
C3	0.8074 (4)	0.42096 (18)	0.6577 (4)	0.0496 (8)	
H3A	0.8368	0.3774	0.7197	0.060*	
C4	0.7993 (4)	0.42102 (15)	0.5027 (3)	0.0397 (7)	
C5	0.8318 (4)	0.35948 (15)	0.4035 (3)	0.0390 (6)	
C6	0.7531 (4)	0.46788 (15)	0.2484 (3)	0.0398 (7)	
C7	0.7534 (4)	0.48617 (15)	0.4094 (3)	0.0399 (7)	
C8	0.7155 (4)	0.55292 (16)	0.4690 (4)	0.0495 (8)	
H8A	0.6854	0.5963	0.4066	0.059*	
C9	0.8028 (4)	0.34924 (16)	0.1157 (3)	0.0447 (7)	
H9A	0.8480	0.2986	0.1496	0.054*	
H9B	0.8919	0.3730	0.0766	0.054*	
C10	0.6092 (4)	0.34476 (15)	−0.0211 (3)	0.0399 (7)	
H10A	0.6228	0.3201	−0.1123	0.048*	
H10B	0.5626	0.3954	−0.0538	0.048*	
C11	0.2381 (5)	0.29295 (18)	−0.1438 (4)	0.0541 (8)	
H11A	0.1381	0.2665	−0.1253	0.081*	
H11B	0.2668	0.2677	−0.2263	0.081*	
H11C	0.1978	0.3435	−0.1773	0.081*	

### Atomic displacement parameters (Å^2^)

**Table:**

	*U*^11^	*U*^22^	*U*^33^	*U*^12^	*U*^13^	*U*^23^
S	0.0537 (5)	0.0383 (4)	0.0401 (4)	−0.0056 (3)	0.0229 (3)	−0.0007 (3)
O1	0.0670 (15)	0.0347 (12)	0.0607 (13)	0.0102 (10)	0.0302 (11)	0.0087 (9)
O2	0.0708 (16)	0.0379 (11)	0.0535 (13)	0.0018 (10)	0.0189 (11)	0.0063 (10)
O3	0.0893 (18)	0.0790 (17)	0.0613 (14)	−0.0205 (14)	0.0507 (14)	−0.0248 (12)
O4	0.0747 (17)	0.0441 (13)	0.0763 (16)	−0.0027 (11)	0.0248 (13)	0.0196 (11)
N	0.0463 (14)	0.0293 (12)	0.0421 (13)	0.0008 (10)	0.0161 (11)	−0.0030 (9)
C1	0.053 (2)	0.0501 (19)	0.077 (2)	−0.0085 (15)	0.0302 (18)	−0.0260 (18)
C2	0.054 (2)	0.072 (2)	0.057 (2)	−0.0098 (17)	0.0289 (16)	−0.0198 (17)
C3	0.0470 (18)	0.0546 (19)	0.0502 (18)	−0.0024 (14)	0.0219 (14)	0.0019 (14)
C4	0.0328 (15)	0.0393 (15)	0.0475 (16)	−0.0028 (11)	0.0158 (13)	−0.0039 (12)
C5	0.0365 (15)	0.0337 (15)	0.0458 (16)	−0.0012 (12)	0.0147 (12)	0.0020 (12)
C6	0.0378 (15)	0.0293 (14)	0.0483 (16)	−0.0010 (12)	0.0117 (13)	−0.0003 (12)
C7	0.0336 (14)	0.0344 (15)	0.0515 (17)	−0.0033 (12)	0.0159 (13)	−0.0065 (12)
C8	0.0469 (18)	0.0367 (16)	0.064 (2)	−0.0022 (13)	0.0199 (15)	−0.0097 (14)
C9	0.0505 (18)	0.0384 (16)	0.0490 (17)	0.0016 (13)	0.0231 (14)	−0.0025 (12)
C10	0.0516 (17)	0.0323 (14)	0.0404 (15)	−0.0016 (12)	0.0227 (13)	0.0002 (11)
C11	0.052 (2)	0.057 (2)	0.0540 (18)	−0.0038 (15)	0.0208 (16)	0.0012 (15)

### Geometric parameters (Å, °)

**Table:**

S—O3	1.430 (2)	C3—H3A	0.9300
S—O4	1.433 (2)	C4—C7	1.394 (4)
S—C11	1.743 (3)	C4—C5	1.490 (4)
S—C10	1.774 (3)	C6—C7	1.483 (4)
O1—C5	1.207 (3)	C7—C8	1.376 (4)
N—C5	1.392 (3)	C8—H8A	0.9300
N—C6	1.404 (3)	C9—C10	1.520 (4)
N—C9	1.457 (3)	C9—H9A	0.9700
C1—C8	1.377 (5)	C9—H9B	0.9700
C1—C2	1.391 (5)	C10—H10A	0.9700
C1—H1A	0.9300	C10—H10B	0.9700
O2—C6	1.210 (3)	C11—H11A	0.9600
C2—C3	1.389 (4)	C11—H11B	0.9600
C2—H2A	0.9300	C11—H11C	0.9600
C3—C4	1.371 (4)		
			
O3—S—O4	116.42 (16)	N—C6—C7	105.8 (2)
O3—S—C10	108.61 (14)	C8—C7—C4	121.5 (3)
O3—S—C11	108.68 (16)	C8—C7—C6	130.2 (3)
O4—S—C10	109.11 (14)	C4—C7—C6	108.2 (2)
O4—S—C11	109.42 (15)	C7—C8—C1	117.4 (3)
C11—S—C10	103.86 (15)	C7—C8—H8A	121.3
C5—N—C6	112.1 (2)	C1—C8—H8A	121.3
C5—N—C9	124.3 (2)	N—C9—C10	113.3 (2)
C6—N—C9	123.5 (2)	N—C9—H9A	108.9
C8—C1—C2	121.4 (3)	C10—C9—H9A	108.9
C8—C1—H1A	119.3	N—C9—H9B	108.9
C2—C1—H1A	119.3	C10—C9—H9B	108.9
C3—C2—C1	120.9 (3)	H9A—C9—H9B	107.7
C3—C2—H2A	119.5	C9—C10—S	112.58 (19)
C1—C2—H2A	119.5	C9—C10—H10A	109.1
C4—C3—C2	117.6 (3)	S—C10—H10A	109.1
C4—C3—H3A	121.2	C9—C10—H10B	109.1
C2—C3—H3A	121.2	S—C10—H10B	109.1
C3—C4—C7	121.1 (3)	H10A—C10—H10B	107.8
C3—C4—C5	130.9 (3)	S—C11—H11A	109.5
C7—C4—C5	108.0 (2)	S—C11—H11B	109.5
O1—C5—N	124.9 (3)	H11A—C11—H11B	109.5
O1—C5—C4	129.1 (3)	S—C11—H11C	109.5
N—C5—C4	105.9 (2)	H11A—C11—H11C	109.5
O2—C6—N	124.4 (3)	H11B—C11—H11C	109.5
O2—C6—C7	129.8 (3)		
			
C8—C1—C2—C3	−0.8 (5)	C5—C4—C7—C8	−179.9 (3)
C1—C2—C3—C4	0.9 (5)	C3—C4—C7—C6	−179.1 (3)
C2—C3—C4—C7	−0.6 (4)	C5—C4—C7—C6	0.7 (3)
C2—C3—C4—C5	179.7 (3)	O2—C6—C7—C8	1.3 (5)
C6—N—C5—O1	−177.4 (3)	N—C6—C7—C8	−179.5 (3)
C9—N—C5—O1	6.6 (4)	O2—C6—C7—C4	−179.4 (3)
C6—N—C5—C4	0.8 (3)	N—C6—C7—C4	−0.2 (3)
C9—N—C5—C4	−175.2 (2)	C4—C7—C8—C1	−0.2 (4)
C3—C4—C5—O1	−3.1 (5)	C6—C7—C8—C1	179.0 (3)
C7—C4—C5—O1	177.2 (3)	C2—C1—C8—C7	0.5 (5)
C3—C4—C5—N	178.8 (3)	C5—N—C9—C10	112.9 (3)
C7—C4—C5—N	−0.9 (3)	C6—N—C9—C10	−62.6 (3)
C5—N—C6—O2	178.9 (3)	N—C9—C10—S	−63.7 (3)
C9—N—C6—O2	−5.1 (4)	O3—S—C10—C9	67.7 (2)
C5—N—C6—C7	−0.4 (3)	O4—S—C10—C9	−60.2 (2)
C9—N—C6—C7	175.6 (2)	C11—S—C10—C9	−176.8 (2)
C3—C4—C7—C8	0.3 (4)		

### Hydrogen-bond geometry (Å, °)

**Table:**

*D*—H···*A*	*D*—H	H···*A*	*D*···*A*	*D*—H···*A*
C1—H1A···O3^i^	0.93	2.34	3.189 (5)	152
C11—H11A···O1^ii^	0.96	2.51	3.463 (4)	175

Symmetry codes: (i) −*x*+1, −*y*+1, −*z*+1; (ii) *x*−1, −*y*+1/2, *z*−1/2.
